# A213 ERCP-RELATED ADVERSE EVENTS IN PRIMARY SCLEROSING CHOLANGITIS: A SYSTEMATIC REVIEW & META-ANALYSIS

**DOI:** 10.1093/jcag/gwab049.212

**Published:** 2022-02-21

**Authors:** N Natt, F Michael, H Michael, S Dubois, A Al Mazrou’i

**Affiliations:** 1 Internal Medicine, Northern Ontario School of Medicine - East Campus, Sudbury, ON, Canada; 2 McMaster University, Hamilton, ON, Canada; 3 Northern Ontario School of Medicine, Thunder Bay, ON, Canada

## Abstract

**Background:**

Endoscopic retrograde cholangiopancreatography (ERCP) is a valuable diagnostic and therapeutic tool in primary sclerosing cholangitis (PSC). Although the complications of ERCP are well known in the general population, there is conflicting data regarding complications in patients with PSC. Factors that predict ERCP-related adverse events in PSC are also unclear.

**Aims:**

To conduct a systematic review and meta-analyses to 1. compare ERCP-related adverse events in patients with and without PSC and 2. determine risk factors associated with ERCP-related adverse events in PSC.

**Methods:**

A systematic search was conducted in Embase, PubMed and CENTRAL for studies published from January 1, 2000 to May 12, 2021. Eligible studies included adults with PSC undergoing ERCP and reported at least one ERCP-related adverse event (bleeding, perforation, pancreatitis, cholangitis) or risk factor associated with complications. Raw event rates for adverse events and risk factors were used to calculate odds ratios (ORs) which were then pooled using random-effects models.

**Results:**

Four studies contributed to the first meta-analysis. There was a significant three-fold increase in the 30-day odds of cholangitis in PSC compared to those without PSC (4.3% vs. 2.0%; OR 3.26, 95% CI 1.08–9.90; *p*=0.037; *I*^*2*^=73.0%) (Figure 1). However, there were no significant differences in 30-day pancreatitis (4.2% vs. 3.4%; OR 0.89, 95% CI 0.26–3.07; *p*=0.851; *I*^*2*^=87.9%), bleeding (0.3% vs. 1.1%; OR 0.36, 95% CI 0.06–2.21; *p*=0.272; *I*^*2*^=50.3%), or perforation (0.7% vs. 0.5%; OR 1.19, 95% CI 0.40–3.51; *p*=0.752; *I*^*2*^=28.5%).

In a second meta-analysis, risk factors contributing to post-ERCP pancreatitis (PEP) in PSC were pooled from five studies. While female sex was not associated with PEP, accidental passage of wire into the pancreatic duct (OR 7.44, 95% CI 3.33–16.65; *p*<0.001; *I*^*2*^=65.0%) and biliary sphincterotomy (OR 4.80, 95% CI 1.92–12.03; *p*=0.001; *I*^*2*^=73.1%) were associated with higher PEP odds.

**Conclusions:**

In the context of limited comparative data and study heterogeneity, PSC patients have higher odds of post-ERCP cholangitis despite the majority receiving antibiotics. Odds of bleeding, pancreatitis, and perforation were similar between groups. Accidental wire passage and biliary sphincterotomy increased odds of PEP, which helps identify higher-risk groups. Future studies should elucidate ERCP-related risks in PSC and guide preventive strategies.

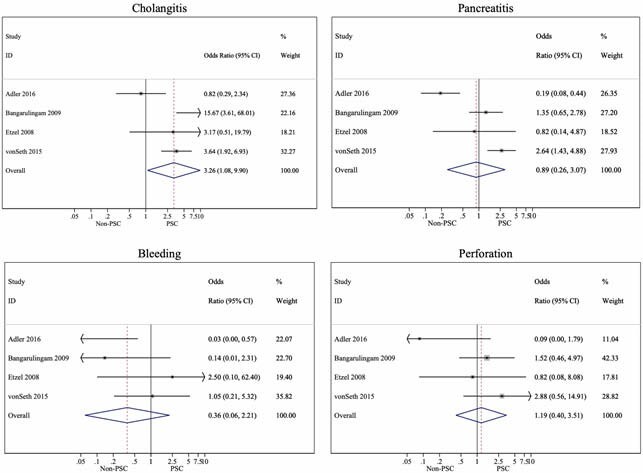

Figure 1: 30-Day ERCP Complications

**Funding Agencies:**

None

